# Suspension of judgment, hinge style

**DOI:** 10.1007/s11229-026-05750-3

**Published:** 2026-08-07

**Authors:** Oscar A. Piedrahita, Sebastian Schmidt

**Affiliations:** 1 Utrecht University, Utrecht, Netherlands; 2 African Institute for Epistemology and Philosophy of Science (ACEPS), University of Johannesburg, Johannesburg, South Africa; 3 University of Zurich, Zurich, Switzerland

**Keywords:** Suspension of judgment, Hinge epistemology, Fallibility, Epistemic justification, Higher-order evidence

## Abstract

In hinge epistemologies, epistemic justification for doxastic attitudes is possible only if a set of assumptions, called hinges, is taken for granted in our inquiries. While hinge epistemologists have focused on how hinges enable the justification of belief, they have not examined whether or how justified suspension of judgment fits within their framework. We argue that any attempt to account for justified suspension encounters two problems, both suggesting that suspension may depend on a hinge of its own. In the positive part of the paper, we show that suspending judgment, like believing and disbelieving, rests on a hinge that renders it both intelligible and justified. We develop the idea that justified suspension about ordinary propositions is explained by taking for granted that one is fallible. This view resolves the identified problems and clarifies how suspension constitutes a legitimate part of hinged epistemic rationality. It also connects hinge epistemology with debates on higher-order evidence and on reasons for suspension of judgment.

## Introduction

Hinge epistemologies (HE) comprise a family of approaches to epistemic justification inspired by the late Wittgenstein’s remarks in *On Certainty* (1969). These approaches characteristically claim that for one to be justified in holding any doxastic attitude toward ordinary propositions, a set of assumptions, also called hinges, must be taken for granted. Consider, for instance, that any information one gathers about events in the distant past cannot serve as a basis for justified beliefs about how things were unless one takes for granted that the Earth has existed for a very long time (Wittgenstein, 1969, Sects. 182–190). Only if the Earth has existed for a very long time can the information, e.g., in a tree ring have “evidential bearing” (Wright, 2004, p. 170) on a proposition about, e.g., past atmospheric conditions and provide justification for a belief in it. These hinges allow us to manage information in our environment as evidentially connected to the objects and facts that our beliefs aim to accurately represent.^1^

Hinges include *very general propositions* taken for granted across all domains of inquiry, such as “one isn’t undergoing radical and systematic deception in one’s beliefs” or “one’s cognitive capacities work mostly reliably”. They also include *slightly less general propositions* assumed in specific domains—for instance, “things don’t disappear when no one is perceiving them” or “there is an external world” in inquiries about all things perceptual; “the world exhibits a multitude of regular, consistent, and predictable phenomena” for inductive beliefs; or the aforementioned “the Earth has existed for a very long time” in astronomical, geological, and historical investigations. Additionally, hinges encompass *particular propositions* required for justification within specific domains, such as “the testifier is a reliable informant on the occasion” in inquiries carried out through testimony (Coliva, 2019), or “there are other minds besides mine” when considering beliefs about other people’s mental states. The central claim of HE is that, depending on the domain, a doxastic attitude is justified only against the backdrop of taking these hinges for granted.

As the examples suggest, HE has focused on hinges and justification *for belief*. But one might think this is somewhat narrow, given that other doxastic attitudes, such as suspension of judgment, are also subject to epistemic rationality.^2^ Consider, for example, how our evaluation of a subject’s suspension about p changes when we learn that she suspended because her evidence for and against p was equally balanced, compared to a case where she suspended because she heard p from a competent, reliable testifier toward whom she harbors racial prejudice. Suspension is justified only in the first case. Some questions immediately arise: Are hinges required for justified suspension, and if so, how? Should we see justified suspension as derivative from justified belief in HE, or does it function independently? What hinges, if any, are involved in justified suspension? These questions deserve attention.

Furthermore, suspending about p typically involves a stance of hesitation regarding the truth or falsity of p, a stance that commentators have articulated in various ways: as an attitude of indecision (Friedman, 2013), resistance to belief and disbelief (Bergmann, 2005), or neutrality (McGrath, 2021; Lord, 2020). This stance requires grounds. Whether one’s suspension is rational or reasonable depends on the quality of one’s evidence and the relationship one has to it—a point emphasized by Wittgenstein through his reflections on doubting (1969, Sect. 4, 122, 310 and ff., 341–342, 456, 458). If HE aims to chart the broader terrain of epistemic rationality, it cannot neglect the justification of suspension. Indeed, understanding how and whether hinges underwrite justified suspension may reveal dimensions of rationality that HE has yet to fully explore.

As important as suspension is to epistemic rationality, not only has HE overlooked the justification of suspension; its framework, we argue, is also seemingly ill-suited to account for it. In Sect. 2, after outlining key ideas from HE, we show that suspension raises two problems, thereby urging proponents of HE to clarify whether and how justified suspension fits their program. The first problem concerns cases in which the domain-specific hinges that ordinarily ground justified belief about p are not taken for granted, yet the subject is justified in suspending about p, which raises the question of which hinge, if any, grounds that justification. The second concerns cases in which a subject’s evidence becomes counterbalanced or equiprobable while the relevant hinges for belief remain operative, which raises the question of how, within HE, justification can shift from belief to suspension. These problems, and the failure of a deflationary approach to justified suspension to solve them, indicate that justified suspension requires its own hinge—one distinct both in content and function from the hinges supporting belief and disbelief. Accordingly, in Sect. 3, we propose that justified suspension is possible thanks to our taken-for-granted fallibility: we’re justified in suspending judgment on non-hinge propositions because we take it for granted that we’re fallible to some degree about them. This proposal, which we connect to recent discussion of the normative import of higher-order evidence (cf. Christensen, 2010; Lord and Sylvan, 2021; Schmidt, 2025; Whiting, 2020), highlights the distinctive epistemic role that our fallibility plays in our rational responses to first-order evidence, and it solves the two problems identified. Section  4 concludes. Overall, the paper aims to show that HE offers key insights into the epistemic structure of justified suspension: just as the justification for belief cannot float free of hinges, neither can justified suspension. This advances the debate within HE and enriches recent discussions on the nature of justified suspension that unfold independently of HE.

## Hinged justification, suspensions, and problems

This section motivates the search for an account of suspension of judgment in HE. Section  2.1 introduces the core claim of HE and discusses whether it should also apply to suspension. Sections  2.2 and 2.3 present the main questions that any HE account of suspension has to answer. Section  2.4 argues against the view that justified suspension derives from (lack of) justified belief. This opens the space for our positive account that suspension has its own hinges, which we develop in Sect. 3.

### Hinge epistemologies’ hinge

The central claim of HE says:

**Core Hinge**: S is epistemically justified in having a doxastic attitude toward p only if S takes a hinge H for granted (where the content of H depends on the domain of p and the method by which p is formed).

The main point is that there cannot be hingeless epistemic justification. A central issue within HE is to explain the relationship, posited by Core Hinge, between hinges and the doxastic attitudes toward ordinary, non-hinge propositions whose justification they enable. How does justification hinge on hinges? Take the hinge that one’s cognitive capacities work reliably. If one were to, for instance, find out whether p by looking, inferring, or taking someone’s word for it, one would gain epistemic justification to believe that p only if one takes for granted that one’s looking, inferring, or taking someone’s word for it are conducive ways of finding out whether p. Imagine one were to find out whether someone is knocking at the door by focusing on any bangs coming from the direction of the door. After hearing three rhythmic bangs, one comes to believe that someone is knocking. But how can one be justified in taking the experience of hearing the bangs as evidence that someone is at the door, without taking for granted that one’s capacities are working properly? After all, it’s by exercising one’s hearing capacities that one rules out that it was just the wind rattling the door and that one comes to believe that there’s someone at the door. If one were to take one’s hearing as defective or unreliable, or if it were an open question whether the bang-like sounds are created by the wind, one might not have a good reason to think that hearing bangs is explained by someone knocking at the door.

What this illustrates is that hinges function as what has been called “norms of evidential significance” (Coliva, 2015, p. 41; Boncompagni, 2024, pp. 291–293). Hinges don’t merely accompany our epistemic practices or supply additional evidence but rather make it the case that some considerations qualify as evidence while others are excluded as epistemically irrelevant within a given inquiry. Hinges fix conditions under which certain experiential inputs can bear on particular propositions at all. To take a hinge for granted in this sense is to treat it as a fixed point that structures inquiry, a certainty from which evidential relations flow. A hinge that is taken for granted in this sense isn’t itself up for assessment within the inquiry it enables; it’s operative in one’s reasoning as a background condition that one does not call into question. This allows it to determine what counts as evidence in the first place. Hinges therefore allow us to overcome our *cognitive locality* (Wright, 2004, pp. 172–174; Coliva, 2015, pp. 3–4). Since experience alone is insufficient to provide justification for propositions beyond our direct awareness (hearing bangs is compatible with nothing but the wind rattling the door; someone could tell one p even when not-p; and a handless dreamer can experience having hands when dreaming), our beliefs about such propositions are justified only if we take for granted that our methods of belief formation, our cognitive capacities, and the information these operate on broadly align with the objects and facts those beliefs are about.

The enabling role of a hinge is not fixed; in certain circumstances, a hinge can become a legitimate object of inquiry. Wittgenstein, for instance, saw the proposition about the existence of one’s hands as a hinge (1969, Sect. 23, 125, 460–461), but noted that it might lose this status if one woke in a hospital and found one’s limbs wrapped in bandages. Similarly, that one’s particular cognitive capacities are mostly reliable can cease to work as a hinge in circumstances where the accuracy of their deliverances is called into question. Consider an optometrist testing one’s vision: in this context, taking the reliability of one’s visual acuity for granted wouldn’t be conducive to epistemic justification to believe in the deliverances of one’s vision, as this is precisely what is under examination. Perhaps one might believe that one’s vision is working properly by taking the optometrist’s word for it. But notice that justification for a belief based on the optometrist’s words hinges on taking for granted that she is a reliable informant on the occasion and, presumably, that one’s capacity to participate in testimonial exchanges and to trust expert testimony works mostly reliably. That is, one must take the workings of the rest of one’s cognitive capacities and the information they operate on to be aligned with the relevant objects and facts, such that one can adjust and correct the rest of one’s beliefs.

Can we apply Core Hinge to justified suspension? Is justified suspension hinged? One may initially resist this suggestion. Belief is a committed doxastic attitude toward a proposition, in the sense that when one believes that p, one’s mental state is such that one (e.g., dispositionally) takes the world to be one way rather than another, namely as if p. The idea of “cognitive locality” already suggests why such an attitude, to be justified, requires taking certain things for granted. Presumably, the world being as if p is not *necessary* for the believer to believe that p*—*otherwise, there wouldn’t be false beliefs. As such, believing is epistemically risky (even if to a minimum degree). Now, if the information that gives one evidence on which to believe p is compatible with not-p, then one’s actual evidence for p is itself insufficient to justify taking the world as if p. To be justified, one needs “collateral information” provided by the hinges (Wright, 2004, p. 170)—one must assume that one’s belief-forming methods, as well as the information they operate on, are broadly aligned with the facts that one’s beliefs aim to accurately represent.

It’s not clear we can say the same of suspension. Unlike belief, suspension doesn’t involve a committed doxastic attitude toward p. It doesn’t involve taking the world to be any particular way regarding p.^3^ It consists instead in withholding commitment, which may require different justificatory conditions than belief. Additionally, if justification of belief is hinged because, all else equal, believing p based on one’s actual evidence carries some epistemic risk, then justification of suspension might not require hinges. Because suspension is a stance of hesitation regarding p, one cannot be wrong about p by suspending. While one might be epistemically wrong in *adopting* a stance of suspension when the evidence warrants belief (especially when p is true), this constitutes being wrong in one’s epistemic stance or attitude toward p, not being wrong *about* p itself. By suspending, one altogether avoids the epistemic risk associated with believing. Thus, one need not take for granted that one’s evidence or basis for suspending is broadly aligned with p. The collateral information provided by hinges seems not to be needed to justify suspension. This might suggest that Core Hinge doesn’t apply to justified suspension.^4^

Treating justified suspension as non-hinged, however, would go against the spirit of HE. HE presents itself as offering a unified account of the structure of epistemic rationality; restricting Core Hinge to belief and disbelief would result in a fragmented picture. We take it then that the hinge epistemologist is pressed to explain how justified suspension is captured by Core Hinge. To this end, we will propose that, to the extent that justified suspension is hinged, we need to think about it in a way that differs from hinged justified belief. Even more, as we’ll see, conceiving justified suspension as hinged in its own distinctive way offers valuable insights into its epistemic structure. Before presenting our positive proposal, however, we’ll introduce two problems that any hinge-based approach to justified suspension must address. Looking at these problems proves instructive.

### The hinge-bind problem

Justified suspension raises two problems, both suggesting that Core Hinge and HE may be inadequate to account for justified suspension. The first we call the *hinge-bind problem*.

Core Hinge claims that justification for any doxastic attitude requires taking a domain-specific relevant H for granted—an assumption that enables justification within a particular method and inquiry, for instance, assuming that perception is generally reliable in perceptual inquiries, or that a speaker is trustworthy in a testimonial exchange (cf. Coliva, 2019). Now, sometimes, a domain-specific relevant H can be neutralized in its role of enabling justification for believing p. This happens when a subject undergoes experiences or receives information with content p, while also having local evidence to not take H for granted (for instance, to not assume that her perception is reliable in the circumstances) and to think that her evidence and experiences as of p are compatible with not-p. Such a subject is typically prevented from having a justified belief that p. Still, she is justified in suspending about p. Yet a question arises for HE: what hinge underwrites this justified suspension, if the domain-specific hinge pertinent to *believing* p isn’t taken for granted in those circumstances? To illustrate, consider these examples.

*Mirrors*: The last thing S remembers is falling unconscious in a house of mirrors, so in her circumstances she can no longer take for granted the H that her perceptual capacities are mostly reliable. S sees a green rectangle in her upper right visual field; it marks the exit from the house of mirrors to the right. S isn’t justified in believing that the exit is to the right. Without further information, S is justified in suspending about whether the exit is to the right.

*Testifiers*: S is in an environment where she receives different pieces of testimony and learns that, for any p, truly believing p from any testifier is as reliable as a coin toss, so S cannot take for granted the H that a speaker is reliable on a given occasion. When S is told p and she lacks independent reasons for either p or not-p, she isn’t justified in believing one way or another. She is nonetheless justified in suspending about p.

If Core Hinge governs justified suspension as it governs belief (and disbelief), there must be a hinge that grounds the justification for suspending about p in these cases. But which hinge does this work? Let’s focus on Mirrors. That hinge cannot be that S’s perception is reliable, for she is justified to suspend on p and yet she doesn’t take for granted that her perception is reliable. Indeed, S is rationally responding to her epistemic situation partly by *not* taking for granted that her perception is reliable. So hinge epistemologists, pressed to be consistent with what Core Hinge predicts (that all justification is hinged), must explain how S can be justified in suspending on p in light of her not taking the relevant hinges for granted (i.e., hinges that would enable justification for a *belief* in p).

Now, there are *other hinges* that S takes for granted and that play some epistemic role in her circumstances. For instance, even if S doesn’t take for granted that her perception is reliable, she still takes for granted that there’s an external world and that her memory is reliable. After all, she wouldn’t be justified in thinking that she is in a house of mirrors unless she takes for granted that her memories of the past are broadly aligned with the facts that those memories are about or that her memories are about a world populated by objects existing independently of her experience of them. Couldn’t these other hinges underwrite S’s justification for *suspending* on p? We don’t think so. The mere presence of these other hinges doesn’t suffice to show that the justification of suspension is hinged. For, presumably, these hinges allow S to form justified beliefs about her evidential situation (e.g., that she is in a house of mirrors, that she remembers falling unconscious in such a place), and to recognize that her evidence is insufficient to settle whether the exit is to the right. But enabling S to assess her evidential situation is not the same as grounding her justification for suspending about whether the exit is to the right. There’s no obvious direct connection between, on the one hand, S’s taking for granted that her memory is reliable and hence being justified to believe that she is in a house of mirrors and, on the other, her being justified *to suspend* on whether the exit is to the right.

Compare this with standard cases of hinged justification of belief. When S forms a justified perceptual belief that there’s a cup on the table, the relevant hinge (e.g., that her perceptual capacities are mostly reliable) directly pertains to the proposition believed. But in Mirrors, the hinges that remain operative pertain to S’s capacity to assess her evidential situation and form beliefs about the past. They don’t pertain to the domain of the proposition about which she suspends. The proposition “the exit is to the right” falls squarely within the perceptual domain, yet the hinge that one’s perception is reliable is not taken for granted. Hinges are configured to ground justified belief within their respective domains. They’re not configured to ground justified suspension about propositions that fall outside those domains.

The hinge-bind problem can then be stated as: when domain-specific hinges for believing p aren’t taken for granted given the local evidence, what hinge grounds the justification for suspending about p? That other hinges that ground beliefs about S’s local evidence are taken for granted doesn’t answer this question. This challenge to hinge epistemology can be formulated as an inconsistent triad:

C1. If no relevant H is in place, S isn’t justified in having any doxastic attitude toward p (Core Hinge).

C2. In Mirrors and Testifiers, no relevant H is in place that grounds a doxastic attitude (e.g., suspension) about p.

C3. In Mirrors and Testifiers, subjects are justified in suspending about p.

C1–C3 cannot be true at once. For hinge epistemologists, rejecting C1 is a non-starter. It’s also unpromising to deny C3. It seems that the hinge epistemologist must reject C2, namely, to argue that, despite appearances, a relevant H is indeed in place in Mirrors and Testifiers. However, rejecting C2 demands an explanation of how there can still be a relevant hinge at work in these cases. This is the hinge-bind problem.

### The shifting attitude problem

A subject can gain evidence that p and, taking the relevant hinges for granted, be justified in believing p; later she gains evidence that not-p and, still taking the same hinges for granted, is justified in believing not-p. How can she suddenly be justified to suspend judgment if her hinges for justification of belief haven’t changed and she simply got additional evidence that favors believing not-p, rather than favoring a distinct attitude of suspension?

At the outset, it’s important to clarify what is not meant to be problematic here. We don’t deny the familiar point that which doxastic attitude is justified depends on a subject’s total evidence, nor that the same piece of evidence can contribute to the justification of different attitudes when combined with different bodies of evidence. Nor do we deny that hinges may remain operative across changes in evidence and thereby play a role in the justification of different attitudes at different times. This much is uncontroversial. The question we wish to press is what resources within HE explain that justification can shift from one attitude to another when total evidence changes.

To better see the problem here, let’s focus on hinge accounts of perceptual justification. According to the conservative framework (Wright, 2004), epistemic justification for a perceptual belief that p requires not only that the subject undergo relevant perceptual experiences *vis-à-vis* p, but also that she have independent justification for taking relevant hinges for granted, such as “there’s an external world” or “I am not dreaming”, etc.^5^ Moderates (Coliva, 2015), by contrast, agree that taking such hinges for granted is necessary for perceptual justification, but deny that an independent justification for these hinges is required. Modeled along the lines of Core Hinge, each approach holds:

*Conservatism*: For any perceptual content p, S is justified in believing p iff, absent defeaters, S undergoes the relevant experience, takes a relevant H for granted, *and has independent justification for assuming/taking H for granted* (Wright, 2004).

*Moderatism*: For any perceptual content p, S is justified in believing p iff, absent defeaters, S undergoes the relevant experience and assumes/takes a relevant H for granted (Coliva, 2015).

Differences aside, both Conservatism and Moderatism uphold Core Hinge: there cannot be perceptual justification without relevant hinges. Notice, for the sake of discussion, that both frameworks specify conditions *for justified perceptual belief*. Neither provides an analogous specification for justified suspension. Now, what we call the “shifting attitude problem” starts with the observation that in cases where S’s evidence is insufficient to support p over not-p, or vice versa, S can be justified to suspend on p. Consider the following cases:

*Counterbalanced Mountains*: At t1, S views a distant mountain range and perceives one peak as the tallest (p). After a change in angle or lighting, S perceives a different peak as the tallest at t2 (not-p). In both instances, S takes the relevant perceptual H for granted (and, if Conservatism holds, let’s assume that S has independent justification for H). Additionally, S has no further information to favor p over not-p.

*Equiprobable Twins*: At t1, it seems to S as if A is in the philosophy department (p) because of some visual perception of someone who looks like A. At t2, S sees A and their twin sibling B, realizing that A has a twin sibling who also works in the same building. At t1 and t2, S’s undergoing such perceptual experience is accompanied by taking the relevant H for granted (and being justified for so doing, if Conservatism holds). At t2, S’s perception makes it the case that the probability that S actually saw A at t1 is 50:50.

Although S is justified to suspend in both cases, there’s still a problem for HE. In both cases, S’s justification at t2 depends on taking for granted the relevant hinge *for a perceptual belief* in p (and not-p). For instance, in Counterbalanced Mountains, the different pieces of evidence at t1 and t2 wouldn’t counterbalance each other unless at those times S took the deliverances of her senses to be broadly aligned with an external world that is accessible to her through perceptual experience. But what explains that in those cases S is justified to suspend on p at t2? After all, in both cases, S’s first-order evidence for p and for not-p doesn’t directly favor suspension of judgment – instead, the evidence that p favors belief, and the evidence that not-p favors disbelief (cf. Schroeder, 2012b, pp. 276–277). Moreover, the hinges that S takes for granted in her circumstances are—according to both Moderatism and Conservatism—*hinges for belief* (or disbelief) in p, *not for suspending* on p. Where does S’s justification to suspend on p at t2 come from, given that at t2 her hinges have not changed and remain hinges for perceptual belief? What accounts for the shift to justified suspension? This is the shifting attitude problem.^6^

One might think that what explains the shift from justified belief to justified suspension is the change in total evidence: that S’s justification to suspend on p is explained by the change in her evidential situation at t2. But a hinge epistemologist cannot rest with this answer alone. Core Hinge requires that any justified doxastic attitude depends on taking relevant hinges for granted. Yet, as the cases above illustrate, there is neither a hinge for suspension nor any other hinge that can explain the shift in justification by appealing to the subject’s total evidence at t2. It’s also far from obvious that the same hinges for belief (which remain untouched at t1 and t2) become hinges for suspension simply by being combined with additional evidence, since, as we explained above, belief and suspension are different kinds of doxastic attitudes (belief, unlike suspension, involves taking the world to be one in which p is the case).

Consider a contrast between two kinds of doxastic shift. When evidence changes and a subject moves from believing p to believing not-p, the same hinge can underwrite both attitudes. The perceptual-reliability hinge, for instance, enables justified belief at t1 and justified disbelief at t2, since belief and disbelief are both committal attitudes, and a hinge that underwrites the epistemic credentials of perceptual experience can ground either one as evidence changes. We cannot say the same for a shift from belief to suspension. A hinge such as *that one’s perception is reliable* enables a doxastic commitment to a perceptual proposition by allowing perceptual experience to count as evidence for taking the world to be a certain way. But suspension involves refraining from taking the world to be any particular way regarding p. Appealing to a change in total evidence doesn’t tell us how a hinge that serves to enable justification for commitment comes to ground justification for withholding commitment.

The shifting attitude problem can thus be formulated as a challenge: when evidence accumulates in ways that shift the justified attitude from belief to suspension, and when the domain-specific hinges for belief remain operative throughout, what explains the justification for suspension? In the positive part of the paper, we will propose that the challenge can be answered by appealing to a hinge so far unidentified, a hinge for suspension. Before that, though, we need to preempt a line of reasoning that might seem to avoid the problems just mentioned.

### How not to close the door

Can we not adopt a deflationary view of justified suspension, according to which being justified in suspending consists in having no justification for either belief or disbelief?

**Deflationary Justified Suspension (DJS)**: S is justified in suspending about p if and only because S has no justification for either believing or disbelieving p (Conee and Feldman, 2004, pp. 83, 102, 179; Comesaña, 2013; Archer, 2024, pp. 38–39).^7^

This view says that a lack of justification for either belief or disbelief explains why suspension is justified. Paired with HE, DJS says that, insofar as justified suspension is captured by the absence of justified belief and disbelief, the hinges for justified belief and disbelief suffice to explain justified suspension. One might think that the hinge-bind and shifting attitude problems rely on an assumption inconsistent with DJS, namely, that justified suspension about p entails more than simply lacking justification for belief or disbelief regarding p.

For instance, the hinge-bind problem suggests that one of the seemingly plausible claims in the C1–C3 triad must be rejected. However, DJS allows for a reinterpretation of these claims that resolves their apparent contradiction. Specifically, one can maintain that no domain-specific relevant hinge directly supports belief or disbelief in Mirrors and Testifiers (thereby upholding C2), while also holding that the neutralization of these hinges itself explains the justification for suspension. This justification for suspension arises precisely because there is no sufficient justification for belief or disbelief based on domain-specific relevant hinges plus the evidence. This interpretation of C2 avoids contradicting C3, which states that in Mirrors and Testifiers, subjects are justified in suspending. At the same time, it doesn’t imply that their justification in these cases is *hingeless*—and it thereby preserves C1 (Core Hinge). Suspension is justified in a hinged manner because the neutralization and insufficiency of the relevant hinges for belief and disbelief determine suspension as the appropriate doxastic attitude.

Similarly, the shifting attitude problem relies on the assumption that hinges and evidence for belief and disbelief end up justifying a different doxastic attitude, namely, suspension. However, under DJS, such a shift cannot occur. What seems to be happening in Counterbalanced Mountains and Equiprobable Twins is that the hinges, as well as the evidence for and against p, are insufficient to justify belief and disbelief in p. Now, since, under DJS, this insufficiency is itself sufficient to justify suspension, these cases don’t involve hinges and evidence for belief and disbelief justifying suspension. Justification for suspension isn’t the result of a hinge doing a double job but rather arises as the rational fallback when the evidence plus the relevant hinges for belief and disbelief fail to provide justification for either of these attitudes.

Notably, this response to the shifting attitude problem is available to both Conservatism and Moderatism.

However, DJS has problems. It implies that there are no direct, positive reasons to suspend, which is far from obvious. We often have positive reasons *for* suspending which don’t just consist in an absence of reasons for belief and disbelief. For instance, knowing that experts in a field genuinely disagree provides a positive reason (at least to non-experts) to suspend. In fact, a number of accounts in the literature articulate different considerations that could directly provide reasons for suspension, such as the costs of error (Schroeder, 2015; Lord, 2020), facts about incoherence (Schmidt, 2023), the risk of false belief (Brunero, 2022), or higher-order evidence (Lord and Sylvan, 2021; Schmidt, 2025). These reasons are supposed to be direct reasons for suspension because rather than *modifying* the weight of one’s reasons for belief, they’re supposed to directly favor suspension just as first-order evidence is supposed to directly favor belief.^8^ Moreover, these considerations might *outbalance* the reasons for belief (i.e., be *weightier* than the reasons for belief) and so explain what it means to lack sufficient reasons to believe (cf. Schroeder, 2021). We think that the hinge epistemologist would do well to remain open to the possibility of direct reasons for suspending. However, we will here commit only to the idea that higher-order evidence provides such direct reasons to suspend, while remaining neutral about these other considerations.^9^

In response to this objection, one might argue that the fact that the evidence is insufficient to justify either belief or disbelief (for instance, when the evidence is equally weighty) is itself a direct, positive reason to suspend. On this view, recently proposed by Avery Archer (2024, p. 77), S is justified in suspending about p whenever S’s “evidence neither conclusively supports the truth or falsity of *p*”. As Archer notes, this approach makes the justification of suspension bear on the state of one’s evidence, which gives one direct reason to suspend.

However, a problem with this suggestion is that it seems to illegitimately double-count the first-order evidence when there is evidence for p and for not-p: *first*, the evidence that p is counted as a reason for belief and the evidence that not-p is counted as a reason for disbelief; *then*, a fact that is constituted by that evidence (for instance the fact that the evidence is evenly balanced or inconclusive) is counted *again* as a separate reason for suspension; yet this fact has *already* been taken into account by saying that the evidence provides reasons for belief and disbelief (cf. Berker, 2018, p. 450; Snedegar, 2017, p. 125; Tucker, 2024, Sect.  3.2). Moreover, it’s notoriously unclear how weighty the reason for suspension is in comparison to the reasons for belief or disbelief. Is it a fixed weight? Does it depend on the strength of the first-order evidence? What effects do factors like practical stakes or higher-order evidence have on the weight of reasons for suspension on this view? These questions suggest that facts about evidence don’t give the most fundamental answer to the question of what kinds of considerations support suspension, and we should instead appeal to other considerations to avoid double-counting and to explain the weight of reasons for suspension.

More generally, DJS is problematic not only on its own but also because it falls short of elucidating the hinged nature of justified suspension. It doesn’t go to the root of the problems. The hinge-bind problem, for instance, is about explaining why the subject’s suspension is justified thanks to relevant hinges when the relevant hinge for belief is neutralized. And the shifting attitude problem requires an explanation of why hinges and evidence for belief and disbelief end up in justification for suspending belief altogether. A deflationary view of suspension’s normative profile leaves these issues untouched. So what are the hinges to suspend?

## The fallibility hinge

Since a deflationary approach to justified suspension isn’t a promising route, we now propose that suspension has its own hinge. This allows us to uphold the claim that all justification is hinged without reducing justification of suspension to (lacking) justification of belief. Return to Mirrors and Testifiers (see Sect. 2.2). As soon as the subject of these cases realizes that she is in an epistemically unfriendly environment, the hinges relevant to the justification of her beliefs are disabled, and so it seems as if she has no reason to hold any doxastic attitude about her environment whatsoever. But if there is a hinge in place that still enables her to gain reasons to suspend judgment, then she could be justified to hold the doxastic attitude of suspension. The same applies to the Counterbalanced Mountains and Equiprobable Twins cases (see Sect. 2.3): if a hinge explains why the subject has reason to suspend, this reason might outweigh the reasons for belief and disbelief.^10^ In this way, a hinge that explains suspension could solve the two problems we’ve identified for HE. In a nutshell, we propose that our fallibility as epistemic subjects grounds the hinged nature of justified suspension:

**Fallibility**: S is justified to suspend, and *only* to suspend, about whether p (absent defeaters) *if and because* S takes for granted that S is fallible to some degree when judging whether p (where “p” can refer to any proposition that is not itself a hinge).

Note that Fallibility is formulated as a sufficient condition and an explanatory claim, not as a necessary condition on justified suspension. Our aim is to identify a hinge that explains why suspension is justified in the problematic cases. Whether Fallibility is the only hinge that can ground justified suspension is a further question on which we remain neutral (we return to this point below).

One’s fallibility as an epistemic subject bears on the rationality of the attitude one must adopt given one’s epistemic circumstances, including the state of one’s evidence. In an HE framework, the justification of belief is a function of the state of one’s evidence, the contents of one’s experiences, and a variety of taken-for-granted hinges that allow one to transcend one’s cognitive locality. Analogously, we propose that justified suspension depends on taking one’s fallibility for granted with regard to specific issues whenever one reasons from the absence of hinges or insufficient evidence for belief to the attitude of suspension. As an account of the structure of justified suspension, Fallibility says that suspension is the *only* doxastic attitude that is justified when one takes for granted that one is fallible about some subject matter *if* that justification is not defeated by sufficient first-order evidence for either p or not-p. That is, neither believing p nor believing not-p is uniquely justified in this case. Note that this view does not imply that suspension is almost always justified. Rather, it implies that suspension is justified only when its justification is undefeated—and in practice, its justification will often be defeated by first-order evidence.

Our view, therefore, says that just as *the Earth has existed for a very long time* and *I am not now dreaming* can be objects of one’s hinge-like attitude, so too can *I am fallible to some degree when judging whether p*. That one is fallible to some degree isn’t something one actively believes or doubts but is itself part of the framework with which we operate as epistemic subjects. Notice that this is the way the recognition of our fallibility works in practice. It’s not as if, after testing the deliverances of our cognitive capacities, each of us *concludes* that we’re fallible beings. Rather, that we’re fallible is woven into different epistemic practices.

Consider practices that involve not merely seeking information we lack, but reconsidering judgments we have already formed: we double-check our conclusions, revisit beliefs in light of new considerations, actively seek counterevidence to positions we hold, and remain open to the possibility that even well-supported beliefs might be mistaken. These practices reflect a recognition of our own fallibility, an openness to the possibility of error, even when we think we have good reason to believe. The baseline assumption that one is fallible is not itself the outcome of an inquiry but part of the framework within which inquiry makes sense.

Of course, experience can provide evidence bearing on *how fallible* we are in particular contexts or with respect to particular tasks. Learning that one is tired, drunk, or biased is evidence that one is fallible *to some higher degree* than one assumes in most ordinary circumstances. Such evidence concerns how fallible one is in a given context, not whether one is fallible at all. In such cases, more or better first-order evidence will be required to defeat the justification of suspension.

In developing this picture of justified suspension in HE, we will employ the language of *epistemic reasons*. We will say that taking for granted some (non-fixed) baseline degree of fallibility about p enables us to gain epistemic reasons for suspending judgment, just as taking for granted that our perception is reliable enables us to gain epistemic reasons for belief. The kinds of reasons that the fallibility hinge allows us to acquire are then provided by higher-order evidence, which is already widely acknowledged as a reason for suspension (e.g., Lord and Sylvan, 2021).

Since we have higher-order evidence for varying degrees of fallibility – as we will explain in detail in Sect. 3.1 below – the fallibility hinge always already comes with an epistemic reason for suspension in one package, so to speak. That is, our hinge-like attitude with the content *that one is fallible to some (non-fixed) baseline degree when judging whether p* renders us eligible to treat higher-order evidence as a reason for suspension, since that higher-order evidence will indicate something about *how* fallible we should take ourselves to be in a specific context. This explains why suspension is justified in the absence of sufficient first-order evidence for either p or not-p.

Evidence that one is fallible to some particularly high degree (that goes beyond some taken-for-granted baseline fallibility implied by Fallibility) when judging whether p provides additional epistemic reason for suspending judgment. By that we mean that if one gains evidence that one is fallible, e.g., by learning about one’s biases regarding p, then more evidence for either p or not-p is required to justify either belief or disbelief in p, i.e., for that evidence to count as *sufficient* evidence. Briefly, epistemic reasons for suspension are provided by a hinge about one’s fallibility and by evidence for one’s fallibility.

This is compatible with there being other reasons for suspension, such as the practical stakes of error (cf. Schroeder, 2012a, b, 2015) or considerations about incoherence (Schmidt, 2023). Plausibly, if the practical stakes of error are reasons for suspension, then they’re enabled by a separate hinge concerning the possibility of error, and maybe another related hinge to the effect that practical stakes matter for epistemic rationality. However, we remain neutral here about other possible hinges for suspension that could enable other kinds of epistemic reasons for suspension. This is why we state Fallibility merely as a sufficient condition and an explanatory claim rather than as a necessary condition on justified suspension. If there are other kinds of hinges for suspension and corresponding reasons for suspending judgment, then even an infallible subject could still be epistemically justified to suspend judgment. This perspective allows for further fruitful discussion in hinge epistemology about hinges for suspension, and how such views could relate to existing accounts of reasons for suspension.

That being said, spelling out Fallibility has its own merits, as this will allow us to solve the two problems we have identified for HE. Doing so requires us to explain the notion of fallibility we have in mind (Sect. 3.1) and what role our fallibility plays in HE (Sects. 3.2 and 3.3).

### Evidence for degrees of fallibility as reasons for suspending judgment

The notion of fallibility at issue here refers to limitations of cognitive capacities when evaluating first-order evidence, rather than the risk of error that is often due to environmental factors, such as fake barns or other kinds of misleading evidence. Our fallibility is most evident in cognitive impairments, such as biases, tiredness, hypoxia, or drunkenness. Facts about how fallible we are right now in forming beliefs about p don’t directly bear on whether p, yet they might prevent us from forming a justified belief about whether p (see Whiting, 2020 for a recent overview of the debate). Evidence for fallibility can have this effect even if our first-order evidence for p is pretty good, we have double-checked many times, and our reasoning is in fact sound.

Consider evidence that one is suffering from hypoxia (Elga, 2008): a condition that impairs reasoning at high altitudes while leaving one feeling confident in one’s judgments. Even if one’s first-order evidence is strong enough to justify belief under normal circumstances, it might not be sufficient to justify belief when one has evidence of being hypoxic. Importantly, since one’s belief might in fact be properly based on strong first-order evidence, evidence of hypoxia doesn’t defeat belief’s justification by weakening one’s first-order evidential support (see Christensen, 2010, p. 195), but by giving one evidence that one’s ability to draw the right conclusions from one’s first-order evidence is impaired. Hypoxia renders belief unjustified, even though it doesn’t directly affect the first-order question of whether p. A plausible explanation is that evidence for hypoxia provides a *pro tanto* reason for suspending judgment (cf. Lord and Sylvan, 2021). This reason can outbalance the weight of one’s evidence for belief.^11^

Another example of evidence for cognitive impairment is evidence that one is biased. Suppose you receive an application from a woman philosopher for a job you have advertised. You know from psychological research that you should consider applications from women more carefully since you’re most likely biased when evaluating their applications (and let’s assume that blinding the application isn’t an option). This evidence from psychological research doesn’t concern the quality of the evidence in front of you (i.e., the application materials), but rather your ability to evaluate this evidence properly, or your tendency to draw wrong conclusions even from properly evaluated evidence. You could be in possession of strong evidence that the candidate isn’t suitable for the next round, but you might still not be justified to believe that the applicant isn’t suitable because you know you are biased. To counteract this bias, you’d have to double-check and ask colleagues for their opinions before you are justified to believe that the application should be rejected. The bias seems to raise the threshold for justified belief. This can be explained by saying that it increases the weight of your total reasons for suspension.

Our proposal is that whenever we attempt to judge an issue that we don’t simply take for granted in our inquiries, we take ourselves to be somewhat fallible with regard to that issue, and therefore have some reason for suspending judgment about it. The central idea is that evidence for conditions like hypoxia or bias is evidence that we’re more fallible than we normally take for granted. That is, we take for granted that we’re fallible to some (non-fixed) baseline degree d_0_ with regard to any proposition that isn’t itself a hinge, but evidence of hypoxia or bias is evidence of a degree of fallibility d_1_, where d_1_ > d_0_. For ease of reference, we call d_0_ our “usual” degree of fallibility, noting, however, that even this baseline degree may vary from proposition to proposition in different contexts, depending on what we take for granted in different circumstances (which is why we call the baseline degree “non-fixed”). The weight of our total epistemic reasons for suspension can then increase when we gain explicit higher-order evidence that we’re more fallible than usual.

Note that there can also be evidence that we’re fallible to some degree *lower* than the degree implied by hypoxia or bias. For instance, we might have some track record in making mistakes when judging where we put our keys. This track record might indicate that we shouldn’t be too confident about this issue, even though our reason for suspension isn’t as weighty as it is when we learn that we’re hypoxic or biased: it’s more easily outweighed by first-order evidence (e.g., a very vivid memory). And yet it also gives us *some* reason for suspension. Evidence that one is hypoxic or biased is just quite an extreme case of this more mundane normative phenomenon whereby degrees of fallibility can provide reasons for suspending judgment, either when we take them for granted or when we possess evidence for these degrees of fallibility.

Lower degrees of fallibility can be quite elusive, which explains why accounts of higher-order defeat have focused on cases where we have strong evidence that we’re fallible to a high degree (i.e., hypoxia or bias). But we suggest that higher-order evidence could give us a very *general* reason for suspending judgment about p, one that we have whenever we possess evidence that we’re fallible to some degree when judging whether p simply due to taking some (non-fixed) baseline degree of fallibility for granted, and so it might be a *ubiquitous* reason (cf. Schmidt, 2025).^12^

This point is central to our proposal: since we might not always *possess* evidence that we’re fallible to some degree—maybe because we have misleading evidence that we’re not fallible, or because we aren’t sufficiently reflective to appreciate evidence about our own cognitive capacities—the view should be complemented by HE: there is some (non-fixed) baseline fallibility that we (i.e., those who participate fully in our practices of epistemic evaluation) take for granted whenever we consider whether p. Suppose someone doesn’t acknowledge their fallibility as a hinge, similar to the king in *On Certainty* who believes that the world began with his birth (cf. Wittgenstein, 1969, Sect. 92). Could this person meaningfully participate in our ordinary epistemic practices? Would they take precautions or update in light of new information? Just as Wittgenstein notes that “conversion” would require the king “to look at the world in a different way”, someone who lacks the Fallibility hinge will fail to acknowledge a set of central reasons for suspending judgment—namely, those provided by our fallibility. Fallibility is revealed through the very structure of our epistemic activities: we deliberate, gather evidence, and revise beliefs precisely because these activities take for granted that we’re fallible beings. This explains why epistemic subjects like us are justified to suspend judgment on propositions not already taken for granted within such practices when we lack sufficient first-order evidence to justify belief or disbelief.

### Fallibility in hinge epistemology: solving the hinge-bind problem

Fallibility states the hinged nature of justified suspension in a way that not only solves the problems articulated in the last section, but is also consistent with a number of familiar ideas among hinge epistemologies.

Starting with the last point, notice that the assumption that our fallibility is taken for granted in our epistemic practices is the flipside of Duncan Pritchard’s *uber hinge*, or one’s overarching commitment to not consider oneself as radically and systematically wrong in one’s beliefs (cf. Pritchard, 2015, p. 99). Indeed, although we cannot be radically and systematically wrong about everything, and so must take certain things for granted, there are still many things that we could be wrong about and so may not take for granted.

In this sense, the hinge epistemological role of our fallibility is also rationally mandated (cf. Coliva, 2015): without taking our fallibility for granted, our practice of epistemic justification as we know it couldn’t get off the ground; too many of our beliefs would count as hinges, since there might not be any general enough reason to suspend judgment about non-hinge propositions (more on its generality at the end of Sect. 3.3), and so there would too often be no need to consider evidence to justify our beliefs in non-hinge propositions. The proposed view thereby illuminates the concept of hinges by understanding them as those propositions where we *don’t* take our fallibility for granted.^13^

Moderate and conservative versions of HE (cf. Sect. 2.3) will spell out this proposal in different ways that reflect their different general conceptions of epistemic justification. On a moderate reading, our proposal would say that justified suspension about p can be explained by the fact that one takes for granted one’s fallibility to some degree and that justification isn’t defeated by first-order evidence for p or not-p. Following Coliva’s version of moderatism, one can say that taking one’s fallibility for granted functions as a constitutive element of our epistemic framework and doing so is mandated by rationality. The conservative account, by contrast, and consistent with its general approach, would require independent justification—or, in Wright’s words, “warrant”—for the fallibility hinge. What would that look like?

Justification to think oneself fallible is available in particular circumstances and is clearly available in cases where one has strong evidence for hypoxia or bias. However, reflective adults also typically possess a substantive awareness of their own fallibility through lived experience, maybe due to having a track record of mistakes in reasoning in certain domains, or to the changing levels of tiredness or focus throughout their daily lives. The conservative need not claim that every subject has such awareness of their cognitive limitations—maybe children and animals are exceptions. But the conservative might claim that one becomes subject to the full range of epistemic evaluations only after one has been initiated into practices that would warrant taking one’s fallibility for granted.

We need not take any position between moderate and conservative implementations of our view since hinge epistemologists can fill in Fallibility according to their preferred account of the structure of epistemic justification. More importantly, let’s see now how our view solves the problems for the justification of suspension in HE.

Recall that the hinge-bind problem can be seen as the tension between three seemingly undeniable claims. Cases such as Mirrors and Testifiers—where subjects lack a relevant domain-specific hinge to justify believing p and yet they’re justified to suspend—suggest the possibility of instances of epistemic justification where no relevant hinge is in place. This would be inconsistent with the core claim of HE. Our view resolves this tension by conceiving justified suspension itself as a type of hinged epistemic justification. This maintains consistency with the core claim of HE. Even in cases where local evidence leaves the subject without a hinge to justify believing p, there remains a hinge to justify suspending belief. In such circumstances, we continue to take some degree of fallibility for granted. This fact explains why we’re justified to suspend whenever we take some degree of fallibility for granted and this justification is not defeated by first-order evidence that supports belief. Our higher-order evidence then modifies the strength of the reason for suspension enabled by our fallibility. (More on the weighing of reasons for suspension against the reasons for belief in Sect. 3.3.) Moreover, our view doesn’t reduce the justification of suspension to a lack of justification for belief and disbelief but acknowledges the role of reasons that might directly favor suspension, the most central of which concerns our fallibility.

This is also the first step toward solving the shifting attitude problem, since suspension can now be justified due to the reason for suspension that can be explained by our fallibility when our first-order evidence for p and not-p is equally strong. However, to fully solve this latter problem, we also need an account of how to determine the weight of reasons for suspension when the normative support shifts from supporting belief to supporting suspension (i.e., when the evidence gets equally balanced). In the next section, we will briefly address this issue by showing how this epistemic reason for suspension interacts with our epistemic reasons for belief that are provided by first-order evidence. This connects hinge epistemology to recent discussions about the weight of reasons for suspending judgment.

### Fallibility and first-order evidence: solving the shifting attitude problem

**Fig. 1:**
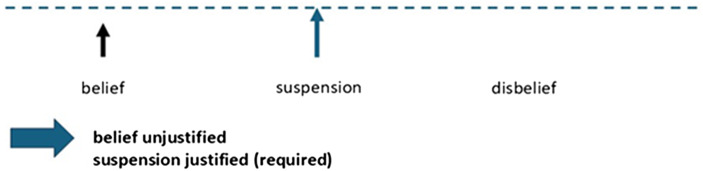
Threshold for justified belief isn’t met

**Fig. 2:**
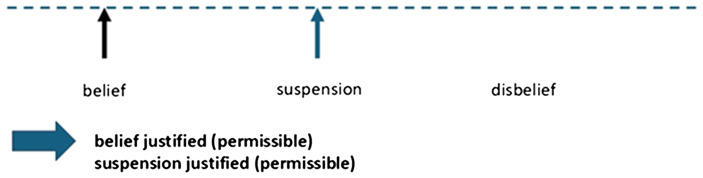
Threshold for justified belief is met

**Fig. 3:**
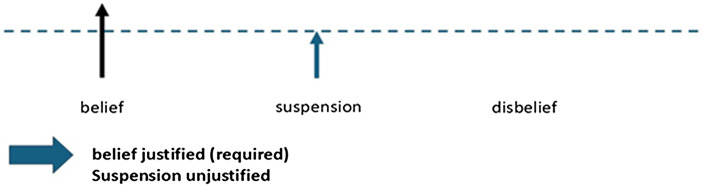
Threshold for justified belief is surpassed

Fallibility implies that we have some *pro tanto* epistemic reason to suspend judgment about all issues that are not hinges. It has this implication because it says that we take for granted some (non-fixed) baseline degree of fallibility that can then be modified by our evidence for fallibility. Any such baseline degree of fallibility will give us at least some normative support for suspension. Since this reason for suspension is *pro tanto*, we’re not normally justified to suspend on ordinary propositions such as whether the animal in front of us is a bird, if the animal is close enough to judge the issue. The reason for suspension that derives from our taken-for-granted degree of fallibility thereby sets a threshold for justified belief, as illustrated in Figs. 1, 2 and 3 below.

The blue arrow over “suspension” represents the weight of the reason for suspension enabled by our taken-for-granted fallibility and provided by our higher-order evidence. The blue dotted lines indicate the threshold for justified belief set by that reason. The black arrow over “belief” represents the weight of the reason for belief provided by first-order evidence that p.

Depending on how weighty that reason is (which in turn depends on the strength of the first-order evidence), it can outbalance one’s reason for suspension. In Fig. 1, we might imagine a case where the bird is still quite far away, so that suspension is still the required attitude (at least once one considers the relevant question). In Fig. 2, the bird is just close enough to justify believing that there is a bird, but we would probably not epistemically criticize someone who still suspends. In Fig. 3, the bird is close enough to demand belief, but it would still be possible to make a mistake, because the proposition isn’t (yet) a hinge.^14^

Note that the reason for suspension is highly variable on the proposed view: concerning many ordinary empirical propositions and under normal circumstances, our taken-for-granted fallibility might be quite low, and so the reason it enables can be easily outbalanced by some first-order evidence. However, if one realizes that one is tired, for instance, one’s total reason for suspension might be weightier, since such evidence for fallibility modifies the weight of the *pro tanto* reason for suspension, and one would thus need more or better first-order evidence that supports belief or disbelief to outbalance this reason. Note that this doesn’t mean that suspension is always justified when one has strong higher-order evidence that one is very fallible due to tiredness, for instance. If one has clear visual evidence or receives testimony from a reliable source, one’s first-order evidence will normally provide a sufficiently weighty reason for belief that renders belief epistemically permissible.

We’re now in a position to address the shifting attitude problem. Recall that the problem arises when evidence accumulates in ways that shift the justified attitude from belief to suspension, while the domain-specific hinges for belief remain operative throughout. The hinges that S takes for granted—perceptual reliability, for instance—are configured to ground committal attitudes such as belief and disbelief. Yet S ends up justified in suspending. What hinge, then, explains or grounds the justification for suspension? Our answer is that the reason for suspending judgment—enabled by our fallibility—is always weightier than the reasons for belief and weightier than the reasons for disbelief when the evidence for p and not-p is counterbalanced. In Counterbalanced Mountains and Equiprobable Twins (see Sect. 2.3), the subject’s justification for suspension doesn’t arise from the same mechanisms that justify belief but from the broader normative standard that suspension is justified when evidence for p and not-p is equally balanced (i.e., provides equally weighty reasons for belief and disbelief).

But why is this reason for suspension always weightier than the reasons for belief when the evidence is equally balanced? Suppose one receives some evidence that not-p in any of the evidential situations in Figs. 1, 2 and 3. Suspension is still the only justified response, while neither belief nor disbelief is justified. Now the reason for suspension will indeed be weightier than the reasons for belief and disbelief in Fig. 1, since here the evidence for p and not-p is rather weak, but it seems that it will be less weighty than the reasons for belief and disbelief in Figs. 2 and 3, since here the evidence for p and not-p is rather strong. However, that cannot be the right result. If suspension is required, then the reason for suspension must be weightier than the reasons for any doxastic alternative in any case of equally balanced evidence. Solving the shifting attitude problem therefore requires an account of why the reasons for suspension are weightier than the reasons for belief and disbelief in Figs. 2 and 3.

A full account of how to weigh epistemic reasons is beyond the scope of this paper and our current aim of addressing the problems about the justification of suspension within HE. This is because *any* account of justified suspension that appeals to positive reasons for suspension (rather than simply deriving justified suspension from the lack of justification for belief and disbelief) must explain why, in cases of equally balanced evidence, the reasons for suspension are always weightier than the reasons for belief and disbelief (cf. Berker, 2018, pp. 449–450). The answer cannot simply be that the reason for suspension increases its weight together with the weight of the reasons for belief—this would seem “like magic” (Berker, 2018, p. 450). Instead, the answer must be found in the normative mechanics of the reasons provided by first-order evidence. We now briefly present a recent account to illustrate what a full solution to the shifting attitude problem might look like and to clarify the role that a reason for suspension based on our taken-for-granted fallibility could play in such accounts.

Chris Tucker (2024) has recently argued that there is some *default reason* for suspending judgment, simply due to the nature of epistemic rationality, and that equally balanced epistemic reasons for belief and disbelief *cancel each other out*, so that the default reason for suspension is *the last reason standing*. Note that a reason enabled by our taken-for-granted fallibility is better suited for HE than a default reason for suspension. A default reason for suspension would erroneously extend suspension even to hinge propositions, whereas our approach reserves suspension for non-hinge propositions. So our approach allows us to transpose Tucker’s view into an HE framework: there is some reason for suspending judgment enabled by our taken-for-granted fallibility with regard to any non-hinge proposition, and since equally balanced epistemic reasons for belief and disbelief cancel each other out, this reason is the last reason standing.

Why do equally balanced epistemic reasons for belief and disbelief cancel each other out? According to Tucker, they do so because they counterbalance in each possible pairwise comparison between doxastic alternatives. Consider the following pairwise comparisons:

- Belief versus disbelief
- Belief versus suspension
- Suspension versus disbelief

In (a), evidence that p is a reason for belief *rather than disbelief*, and evidence that not-p is a reason for disbelief *rather than belief*. In (b), evidence that p is a reason for belief *rather than suspension*, and evidence that not-p is a reason for suspension *rather than belief*. In (c), evidence that p is a reason for suspension *rather than disbelief*, and evidence that not-p is a reason for disbelief *rather than suspension*. Since the reasons coming from first-order evidence counterbalance in each pairwise comparison, they cancel each other out. In contrast, the reason for suspension enabled by our taken-for-granted fallibility doesn’t counterbalance in each pairwise comparison, since it doesn’t bear on the comparison between belief and disbelief at all. Suspension therefore becomes the rational attitude—the evidence is insufficient to justify (dis)belief and the fallibility hinge provides a positive reason to suspend. This remaining “last reason standing” then explains why subjects rationally *shift* from belief/disbelief to suspension under conditions of equiprobable or counterbalanced evidence, since the reasons provided by this evidence cancel each other out.^15^

We don’t commit to Tucker’s specific view here. Instead, we note that recent literature on the reasons for suspending judgment has provided explanations for why reasons for suspension are always weightier than those for belief or disbelief in cases of equally balanced evidence (cf. also Vollmer, 2024; Schmidt ms). Tucker’s framework illustrates how a non-evidential reason—such as the one enabled by the fallibility hinge—can fulfill this explanatory role by serving as the last reason standing after first-order evidential reasons have canceled out.

Our proposal thus integrates smoothly with HE’s central commitments by grounding justified suspension in a distinct hinge—our taken-for-granted fallibility—that applies exclusively to non-hinge propositions. This approach avoids the problematic implication of having default reasons to suspend on hinges themselves. Instead, it clarifies that genuine hinges are precisely those propositions about which we don’t take our fallibility for granted and consequently have no positive reason to suspend judgment.^16^

By introducing fallibility as a distinct hinge, our account solves the shifting attitude problem. Since it enables higher-order evidence as a reason for suspension, it allows us to gain an independent, positive reason justifying suspension when first-order evidence is equally balanced. Unlike deflationary approaches that merely equate suspension with a lack of justification for belief or disbelief, our account avoids double-counting first-order evidence and integrates naturally with the core claim of hinge epistemology.

## Conclusion

We have proposed an account of justified suspension for hinge epistemology. First, we have argued that HE is seemingly ill-suited to account for justified suspension. The hinge-bind problem arises when domain-specific hinges for belief about p are not taken for granted, yet the subject is justified in suspending about p, which raises the question of which hinge, if any, grounds that justification. The shifting attitude problem arises when a subject’s evidence becomes counterbalanced while the hinges for belief remain operative throughout, which raises the question of how, within HE, justification can shift from belief to suspension when the hinges in place are configured for committal attitudes. We have argued that a deflationist approach that reduces justified suspension to the lack of justification for belief and disbelief isn’t promising. Instead, we have argued that justified suspension is grounded in a distinct hinge that appeals to our taken-for-granted fallibility. This provides a framework for HE that accounts for justified suspension while remaining consistent with HE’s core claim that all justification is hinged.

It’s worth noting that our proposal invites further exploration of the role of fallibility as a hinge and how it interacts with other hinges in structuring epistemic rationality. If justified suspension depends on the Fallibility hinge, then this challenges and refines the existing understanding of the structure of epistemic commitments within HE frameworks. Moreover, our discussion opens the space for debate about the normativity of suspension in hinge epistemology more generally. While we have identified Fallibility as one central hinge for suspension that enables fallibility-related reasons for suspension, we left it open whether there are other kinds of hinges for suspension that could enable different kinds of reasons for suspension (such as practical stakes or incoherence-related reasons). HE is only at the beginning of exploring these issues, and it can draw inspiration from other contemporary debates about epistemic reasons and the normativity of suspension.

## Data Availability

Not applicable

## Footnotes

1 One issue we set aside concerns the ontological status of hinges. Some view them as propositions (Wright, 2004; Coliva, 2015; Pritchard, 2015) and others as non-propositional rules (Moyal-Sharrock, 2004, 2022). It’s also highly contested what “taking a hinge for granted” entails. Doxastic accounts interpret the attitude toward hinges as acceptance (Wright, 2004), a non-evidential propositional attitude (Coliva, 2015, 2022), or belief (Kusch 2016a, b; Barranco López 2023), while non-doxastic accounts describe it as a *sui generis* propositional commitment (Pritchard, 2015) or as an animal way of acting (Moyal-Sharrock, 2004, 2022). A further debate concerns the epistemic status of hinges or our attitude toward them: they’re variously described as non-justified and a-rational (Pritchard, 2015; Moyal-Sharrock, 2004, 2022), rational though not justified (Coliva, 2015; Piedrahita, 2021), evidentially justified (Kusch 2016a, b; Neta 2021; cf. Williams, 2007), justified non-evidentially (Wright, 2004), or even as a form of knowledge (Engel, 2015; Greco, 2021). For present purposes, we use “taking a hinge for granted” broadly to capture the attitude specified by one’s preferred HE, while bracketing questions about hinges’ epistemic support and ontology.

2 To keep things manageable, we concern ourselves with outright doxastic attitudes rather than degrees of belief, and we assume that there are precisely three doxastic alternatives, where suspension is a doxastic attitude that others label as “agnosticism” (Wagner, 2022; Archer, 2024) or “withholding judgment” (e.g., Schroeder 2012b, 2015; Tucker 2024). We must bypass these debates here, as well as accounts of the precise nature of suspension, and simply note that our view isn’t obviously in conflict with these views.

3 We’re not ruling out views for which suspension constitutively involves, or is a species of, belief (e.g., a belief about one’s beliefs, or about one’s epistemic situation, or about the state of one’s evidence (cf. Crawford, 2004; Bergmann, 2005; Masny, 2020; Raleigh, 2021). For critical discussion of such views, see Archer (2024, ch. 3).

4 Core Hinge is a generalization of the central animating idea behind HE—namely, that epistemic rationality requires taken-for-granted hinges—rather than a claim that hinge epistemologists have explicitly endorsed about, say, suspension. While the literature has focused primarily on belief, formulations of HE are naturally read as governing epistemic rationality or justification in general. Wittgenstein himself emphasized that “the questions that we raise and our doubts depend on the fact that some propositions are exempt from doubt, are as it were like hinges on which those turn” (1969, Sect. 341). If the rationality of attitudes like belief, doubting and questioning depends on hinges, then we should expect suspension, as a doxastic attitude, to be covered by the hinge framework from the outset.

5 Wright talks in terms of independent ‘*warrant*’, which can be either evidential or non-evidential, the latter being characterized as *entitlements*.

6 Note that this is not a problem that we have just invented. Rather, the literature on reasons for suspension contains a parallel problem. According to Schroeder, for instance, reasons to “withhold belief” cannot be (first-order) evidence “because the evidence is exhausted by evidence which supports p and evidence which supports ~ p. But the evidence which supports p is reason to believe p, and the evidence which supports ~ p is reason to believe ~ p. Consequently the reasons to withhold must come from somewhere else. So they cannot be evidence” (Schroeder 2012b, pp. 276–277). We’re here spelling out the shape this problem takes within a hinge framework. The general problem is this: how do we explain justified suspension if suspension is its own distinct kind of doxastic attitude? The specific problem in terms of reasons is: what kinds of considerations justify suspension? Our specific problem in hinge epistemology is this: what do we need to take for granted so that suspension can be justified under certain evidential circumstances? As we will see, our account will also offer an (at least partial) answer to the question in terms of reasons, since we hold that evidence for fallibility contributes to suspension’s justification by providing reasons for suspension.

7 DJS also follows from a deflationary view of *the nature* of suspension, one for which suspension about p is merely the absence of belief and disbelief in p (Chisholm, 1978, p. 177). This view has been well criticized in the literature. So we don’t discuss it in the main text. For one, lack of belief and disbelief is not sufficient for suspension (Friedman, 2013, p. 168 and ff.). S might lack belief and disbelief in p for various reasons, such as being the wrong kind of subject (e.g., a baby or a table), never considering p, or not grasping p. However, in none of these cases does this lack result in suspension or a stance of hesitation. Moreover, lack of belief and disbelief isn’t necessary to suspend, given the possibility of inconsistent doxastic attitudes. It’s possible to believe p and suspend on p, just as one might believe both p and not-p. While such a state might be incoherent or irrational, nothing inherent in the nature of suspension excludes believing or disbelieving the same proposition one suspends about (Friedman, 2017, p. 305; Atkins, 2017, p. 3041; Archer, 2024, p. 12 and ff.). Besides Friedman (2013, 2017), others rejecting this deflationary view include Bergmann (2005), Atkins (2017), McGrath (2021), Raleigh (2021), Archer (2024, ch. 2). Note that, given our purposes, we need not subscribe to any specific non-deflationary account. For a useful survey and critical discussion of various non-deflationary views of suspension, see Archer (2024).

8 On direct and indirect normative support, cf. Tucker (2025﻿). The basic idea is that considerations that bear indirectly on the justification of an attitude *modify* or *attenuate* or *enable* the weights of other kinds of reasons that directly support an attitude.

9 For a discussion of advantages of the view that higher-order evidence provides a very central reason for suspension as compared to stakes of error, risks of error, and incoherence-based reasons, see Schmidt (2025, pp. 237–238).

10 We return to the question of why this reason is always weightier than equally balanced reasons for belief and disbelief in Sect.  3.2.

11 The reason for suspension coming from higher-order evidence is *pro tanto* because it need not outbalance one’s reasons for belief. Indeed, first-order evidence can be so strong that any evidence that one is cognitively impaired—e.g., tired or drunk—doesn’t matter much, as when an overworked doctor, who is therefore also bad at diagnosing, is still rationally confident in her particular diagnosis that a patient’s hair is on fire (cf. Weatherson, 2019, p. 131).

12 Schroeder (2012a) famously argues that state-given reasons, such as stakes of error and the availability of further evidence, are ubiquitous. For more about ubiquitous epistemic reasons, see the last paragraph of Sect.  3.3 below.

13 This makes HE immune against traditional versions of radical skepticism, since we don’t need any epistemic reason for believing p to be justified to believe p (say, that there is a tree in front of me) if there is no epistemic reason for any other alternative to belief (i.e., no reason for suspending judgment about p or for disbelief); but skepticism could still be articulated in pragmatic terms, i.e., by appealing to high stakes of error as strong and general reasons to suspend judgment. But then skepticism calls for a pragmatic response (see, e.g., Ranalli, 2024, Rinard, 2021).

14 If you don’t believe in positive epistemic duties to believe (but see McGrath, 2025; Simion, 2024; Ichikawa, 2024), then you will think that the case depicted in Fig. 3 is not possible. Our core view is flexible enough to allow such theoretical restrictions arising from other epistemological considerations. We largely wish to stay neutral here.

15 Tucker’s view assumes a version of *contrastivism* about epistemic reasons (cf. Snedegar, 2017: ch. 6), according to which first-order evidence that not-p can be a reason for suspension *rather than* a reason for belief but not a reason for suspension *full stop*.

16 Could there be reasons to suspend judgment about hinges? Maybe in certain philosophical contexts, as when we raise radical skeptical doubts, we might have some philosophical or metaphysical doubt about hinges. In general, it’s always in principle possible for hinges to lose their status as hinges even in our daily life. Cf. also Wittgenstein (1969, Sect. 97). That’s not a problem for our proposal, however. For insofar as we take ourselves to be fallible about a hinge, to that extent we will also no longer treat it as a hinge. Thanks to an anonymous referee for pushing us to make this explicit.
